# Covid-19 Has Turned Home Advantage Into Home Disadvantage in the German Soccer Bundesliga

**DOI:** 10.3389/fspor.2020.593499

**Published:** 2020-11-05

**Authors:** Markus Tilp, Sigrid Thaller

**Affiliations:** Institute of Human Movement Science, Sport and Health, University of Graz, Graz, Austria

**Keywords:** team performance, referee home bias, familiarity, travel fatigue, crowd support

## Abstract

The main factors for home advantage (HA), quantified by the number of points won at home expressed as a percentage of all points, are believed to be crowd support, territoriality, familiarity, and travel fatigue. In 2020, the German Soccer Bundesliga interrupted its championship due to the Covid-19 pandemic after 25 rounds and the last nine rounds were played without audience. This unique situation allowed studying the effect of spectators on the team's performance and the referee's decisions. We hypothesized a decrease in HA and a more balanced distribution of fouls and disciplinary cards in the games without audience (GWOA) compared to the games with audience (GWA). We evaluated *n* = 223 GWA and *n* = 83 GWOA of the season 2019/20 and all games of the preceding season 2018/19 to analyze the distribution of game outcomes (wins, losses, and draws) and HA. We analyzed the number of fouls, disciplinary cards, and penalty kicks. We found significant differences in HA between GWA (HA = 54.35%) and GWOA (HA = 44.1%) as well as GWOA and games of 2018/19 (HA = 57.63%). The distribution of game outcomes in GWOA did not differ from GWA but differed significantly from 2018/19 (*p* = 0.031). The distribution of fouls showed a significant difference to equal distribution in GWA [home: 2,595 (48.56%); away: 2,749 (51.44%)] but not in GWOA [home: 1,067 (50.54%); away: 1,044 (49.46%)]. In the GWOA, we counted 178 (51.1%, home) and 170 (48.9%, away) cards, representing a significant difference in the distribution to GWA [home: 405 (44.85%); away: 498 (55.15%)]. The number of red cards differed significantly from an equal distribution for GWA (14 home and 28 away) but not for GWOA (eight home and seven away). In the last nine rounds without audience, we observed more home losses (36) than home wins (27). Hence, the Covid-19 lock-down led to a home disadvantage. One reason for this surprising result could be that the home team is missing an important familiar aspect when playing in their empty stadium without social support from their home audience. Furthermore, both teams know about the HA thus the away team could be more motivated in this unusual situation.

## Introduction

Association football (soccer) is one of the most popular team sports in the world. Depending on the country and the league, soccer games attract a large audience. In the German Soccer League (Bundesliga) season 2018/19, the average number of visitors was about 43,000. The largest stadium, the Signal-Iduna Park in Dortmund, has a capacity of more than 80,000 spectators. It is reasonable that the presence of such a big audience supporting the home team affects the players and referees in favor of the home team.

Home advantage (HA), quantified by the number of points won at home expressed as a percentage of all points at home and away (Pollard, 1986; Pollard and Gómez, 2014b, 2015), is a well-studied phenomenon in soccer. Despite a decline in the last decades, probably due to increased professionalism, distancing of players from fans, and globalized marketing strategies that decreased the identification of the local community with clubs (Smith, 2003; Pollard, 2006a), HA is still present in professional soccer (Peeters and van Ours, 2020). A crucial assumption for calculating the HA is a balanced schedule, meaning that all teams play two games against each other, one at home and one away (Pollard and Gómez, 2014b). In the last 50 years, many authors tried to find the main factors explaining why the home teams on the average score better than the away teams (see e.g., Nevill and Holder, 1999; Pollard, 2008 for review). The HA's main factors are believed to be crowd support, territoriality, familiarity with the stadium, and travel fatigue (Ponzo and Scoppa, 2018).

Several aspects of crowd support were investigated in literature: Nevill et al. (1999, 2002) examined the crowd noise and found effects on referee decisions. Pollard and Pollard (2005) investigated the effect of crowd size but found only small effects on HA, similar to previous reports (Dowie, 1982; Pollard, 1986; Clarke and Norman, 1995). Several researchers found evidence for a referees' bias in terms of fouls, disciplinary cards, and awarded penalty kicks during games (Nevill et al., 2002; Garicano et al., 2005; Boyko et al., 2007; Dohmen, 2008; Dawson and Dobson, 2010). Home crowd support seems to put significant social pressure on referees leading to biased decisions. Referees who observed soccer videos awarded less fouls to the home teams when the crowd noise was played than when the clips were played in silence (Nevill et al., 2002). Similarly, Unkelbach and Memmert (2010) observed that referees awarded more yellow cards to the away team when videos with home crowd noise were played with high compared to low volume. Furthermore, Sutter and Kocher (2004) reported that officials grant more injury time after 90 min when the home team is behind one goal compared to matches in which it leads by one goal at the end of a game. This so-called ±1 bias could be confirmed by other authors (Garicano et al., 2005; Riedl et al., 2015). A similar bias could also be observed for big teams by Lago-Penas and Gómez-López (2016), who reported that referees tended to add more extra time when the higher-level team was behind and less extra time when the team was ahead in close games. Interestingly, Albanese et al. (2020) reported recently that the introduction of additional assistant referees reduced referee bias in favor of home teams. Pollard (2006b) also reported regional differences in HA with values from 49 to 79% and particularly high values in the Balkans and the Andean countries of South America. They suggested that this is related to high levels of territoriality in these countries, a concept that was first mentioned by Morris (1981). Evidence for this was also found on an individual physiological level when players of the home team had increased testosterone values (Neave and Wolfson, 2003, 2004; Wolfson and Neave, 2004). Home advantage as a social phenomenon (Schwartz and Barsky, 1977) was confirmed by Gelade (2015). He could show that HA is elevated in countries with high levels of collectivism and in-group favoritism as well as in countries with high levels of corruption and where the rule of law is not strictly adhered to. Social support by the audience increases performance of the home team (Salminen, 1993) and even inhibits the performance of the away team as reported from college basketball games by Greer (1983). As to the familiarity to the stadium, there are, on one hand, physical properties as for example the temperature/altitude, the pitch surface, and the pitch size (Pollard, 1986; Barnett and Hilditch, 1993; Clarke and Norman, 1995). On the other hand, there are psychological familiarity and perception (Bray et al., 2000) of the players (see Neave and Wolfson, 2004 for review), the “feeling at home,” which is in turn also influenced by the crowd.

Furthermore, travel fatigue may occur when the locations of the different games are far apart (Pollard et al., 2008; Pollard and Gómez, 2014a). During same-stadium derbies, where the effects of travel fatigue or familiarity with the stadium are not present but the level of crowd support still differs, conflicting results have been reported. While Ponzo and Scoppa (2018) still observed a HA, this was not the case in same-stadium derbies analyzed by van de Ven (2011).

Altogether, the different factors are not independent but are related to each other. Statistical calculations of large data sets over many countries and years show some relations (Pollard and Gómez, 2014a). Despite the clear quantitative evidence of HA in soccer, the different factors' proportionate effects are not clear because it is not possible to undertake relevant experiments without audience in professional soccer.

To the best of our knowledge, until 2020 only one study so far included professional soccer games without audience in their analyses of HA (van de Ven, 2011). However, this study only included twenty games of the Italian Series A(7) and B(13) (Season 2006/2007), which only allows limited statistical analyses. Due to the restrictions during the Covid-19 pandemic in 2020, many soccer leagues worldwide had to play without audience. Hence, several working papers have recently been published that analyzed the effects of these so-called “ghost games” on home performance and referee decisions. Fischer and Haucap (2020) analyzed the first three divisions of the German Bundesliga and observed a reduction of the HA only in the first league which was related to occupancy rates, i.e., HA was reduced most in teams with high occupancy in their stadium during regular seasons. McCarrick et al. (2020) analyzed the effect of games without audience collapsed from 15 different European soccer leagues and found that empty stadiums decreased home team performance and affected referees' decisions. When analyzing 23 leagues worldwide, Bryson et al. (2020) discovered large and statistically significant effects on the number of yellow cards when no audience was present. Referees issued fewer cards to away teams which reduced the home advantage. This reduction in social pressure for referees was also confirmed by (Endrich and Gesche, 2020) who reported that home teams were treated less favorable during the games without audience in the first and second German soccer league.

In 2020, the German Soccer Bundesliga interrupted its championship in March due to the Covid-19 pandemic after 25 of 34 rounds. The championship continued in May, but the last nine rounds had to be played without audience. Hence, this unique situation allowed studying the effect of spectators on the team's performance and referee's decisions in professional soccer.

Based on the well-established effects of crowd support on HA, we hypothesized that the lack of audience would (a) decrease HA and (b) lead to a more balanced distribution of disciplinary cards, penalty kicks, and fouls by the referees in the games without audience (GWOA) compared to the games with audience (GWA) in the 2019/20 season and the 2018/19 season, respectively. Furthermore, we hypothesized (c) differences in the distribution of home wins, home losses, and draws between the games with and without audience.

## Method

### Sample

In total, we evaluated all 306 soccer games of the season 2019/20, 223 with audience (25 rounds with nine games each minus two postponed games from round 22 and 24 without audience) and 83 without audience (nine rounds plus the two postponed games). In addition, we retrieved the results of the preceding season 2018/19 for further comparison of distributions with a balanced playing schedule. For assessing the referees' bias, we evaluated 7,455 fouls, 1,251 yellow cards, 57 red cards, and 72 penalty kicks.

### Procedures

For all games, we counted the home wins, the home losses, and draws. We calculated the HA as the ratio between the number of points won by the home teams and the total number of points (Pollard, 2008; Pollard and Gómez, 2014b).

The comparison between the outcomes of games of an unbalanced playing schedule could produce a bias, e.g., if more better teams would have had more away games in one group of games compared to the other. Therefore, to assess the effects of the unbalanced playing schedule, we first calculated the percentage of games without audience, where the home team was ranked higher than the away team according to the final table ranking of the 2019/20 season.

However, the ranking alone does not determine uniquely the probability of the awarded points in a game. Therefore, for a rough estimation of the probability for possible results in each game we applied a Poisson distribution (Maher, 1982; Greenhough et al., 2002) based on the average number of goals per game during the whole season as the expected rate of occurrences. For each game, we first calculated the probability of the number of goals shot in this game for both teams via the Poisson formula:

$$\begin{array}{l} p_{s}\left(a\right)\;=\;\frac{a^{s}}{s!}e^{-a}, \end{array}$$

where *a* denotes the average number of goals, *p*_*s*_ *(a)* the probability of shooting *s* goals.

The probability of getting a result of *s:t* for two teams with the average number of goals *a* and *b*, respectively, is given by multiplying the single probabilities for each team:

$$\begin{array}{l} p_{s,t}\left(a,b\right)\;=\;\frac{a^{s}}{s!}e^{-a}\cdot \frac{a^{t}}{t!}e^{-b}. \end{array}$$

Then we calculated the probability of each possible result of a game (up to a result of 8:8, which is already very unlikely in soccer). Counting the wins, draws, and losses, we got the expected percentage of home wins, home losses, and draws. We computed the expected number of points for the home team and the away team from this distribution. As an example, we show the procedure for team A (Eintracht Frankfurt) and team B (SC Freiburg). The average number of goals for the two teams were 1.74 and 1.41 according to the results at the end of season 2019/20. Table 1 shows the probability of each outcome of the game.

**Table 1:** Predicted results of an exemplary game between team A (Eintracht Frankfurt) and team B (SC Freiburg): The value in the m^th^ line and the n^th^ column shows the probability for the result m:n.

**A**\**B**	**0**	**1**	**2**	**3**	**4**	**5**	**6**	**7**	**8**
0	0.043	0.0607	0.0428	0.0202	0.0071	0.002	0.0005	1E-04	2E-05
1	0.0746	0.1053	0.0743	0.035	0.0123	0.0035	0.0008	0.0002	3E-05
2	0.0647	0.0914	0.0645	0.0303	0.0107	0.003	0.0007	0.0001	3E-05
3	0.0374	0.0528	0.0373	0.0176	0.0062	0.0017	0.0004	8E-05	1E-05
4	0.0162	0.0229	0.0162	0.0076	0.0027	0.0008	0.0002	4E-05	6E-06
5	0.0056	0.008	0.0056	0.0026	0.0009	0.0003	6E-05	1E-05	2E-06
6	0.0016	0.0023	0.0016	0.0008	0.0003	8E-05	2E-05	4E-06	6E-07
7	0.0004	0.0006	0.0004	0.0002	7E-05	2E-05	4E-06	9E-07	2E-07
8	9E-05	0.0001	9E-05	4E-05	1E-05	4E-06	1E-06	2E-07	3E-08

This results in the probabilities 0.45– 0.31–0.23 for home wins, home losses, and draws, leading to 1.59 points for the home team and 1.18 points for the away team.

We did this for all GWOA and got an expected HA, again dividing the home teams' total points by the sum of the total points of home teams and away teams.

For assessing a possible referee's bias, we investigated three indicators: Firstly, we counted the number of awarded fouls. The distributions for home teams and away teams were compared to a 50:50 relation for GWA as well as for GWOA. Secondly, we counted the number of disciplinary cards (yellow and red) for both teams and calculated the ratio between home cards and all cards. The ratios of home team disciplinary cards for the GWA and GWOA were compared to a 50:50 relation, respectively. Furthermore, we tested for the difference in the number of cards per game and fouls per game. As third indicator of a referee's bias, we analyzed the number of awarded penalty kicks for the home team and the away team.

### Statistics

We compared the HA and the distribution of home wins, home losses, and draws for 233 GWA and 83 GWOA to the expected distribution got from the 306 games in the preceding season 2018/19 by applying chi-square goodness of fit tests. Furthermore, we compared the HA and the distributions of home wins, home losses, and draws between the 83 GWOA and the 223 GWA by applying chi-square goodness of fit tests.

We compared the expected HA calculated by Poisson distribution to the observed HA again using a chi-square goodness of fit test.

In addition, the ratio of cards and the ratio of fouls for GWOA were compared to GWA, again by chi-square tests for the goodness of fit. As there were only a few red cards, the difference in the percentage of red cards was assessed by a binomial test. As an unbalanced playing schedule does not affect the referee's decisions, it was not necessary to compare the results of GWOA with previous seasons. However, we tested differences in the average number of fouls per game between GWA and GWOA for home and away teams with *t*-tests.

We compared the ratio of awarded penalty kicks for the home team and the away team between GWA and GWOA and to a 50:50 distribution applying chi-square tests for the goodness of fit.

In all tests, significance was defined for *p* < 0.05.

## Results

**Figure 1:**
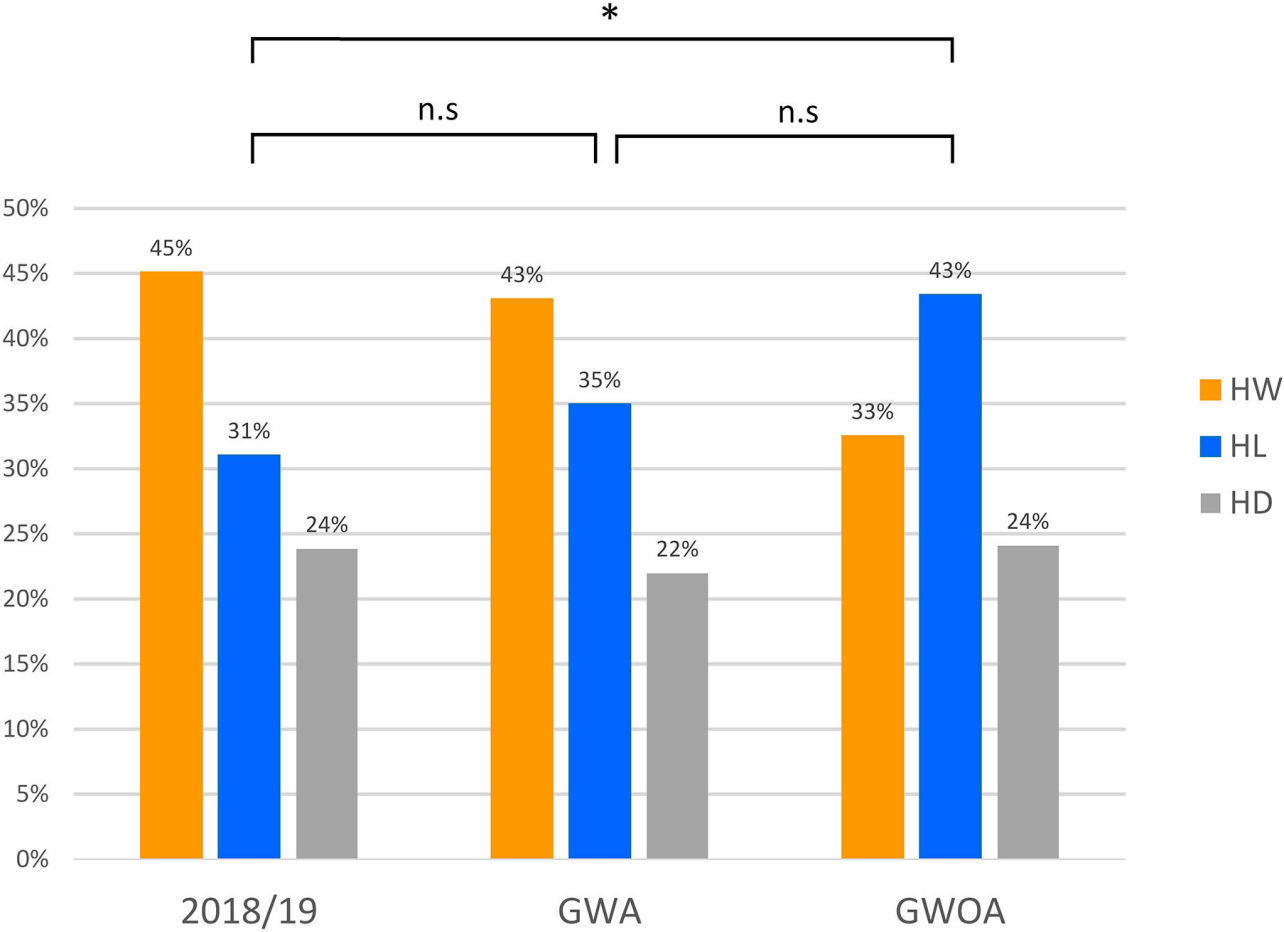
Distributions of home wins (HW), home losses (HL), and draws (HD) for the season 2018/19 (with audience), and games with (GWA) and without (GWOA) audience for the season 2019/20. * indicates significant difference in chi-square goodness of fit (*p* < 0.05). n.s. indicates no significance.

Figure 1 shows the distribution of home wins, home losses, and draws in the GWA, GWOA, and the season 2018/19. In the 83 GWOA, we observed 27 home wins, 36 home losses, and 20 draws, which did not differ (*p* = 0.137) from the expected distribution observed in the GWA, but differed significantly from the season 2018/19 (*p* = 0.031). In the 223 GWA, we observed 96 home wins, 78 home losses, and 49 draws, showing no significant difference to the games of the previous season (138 home wins, 95 home losses, 73 draws).

**Figure 2:**
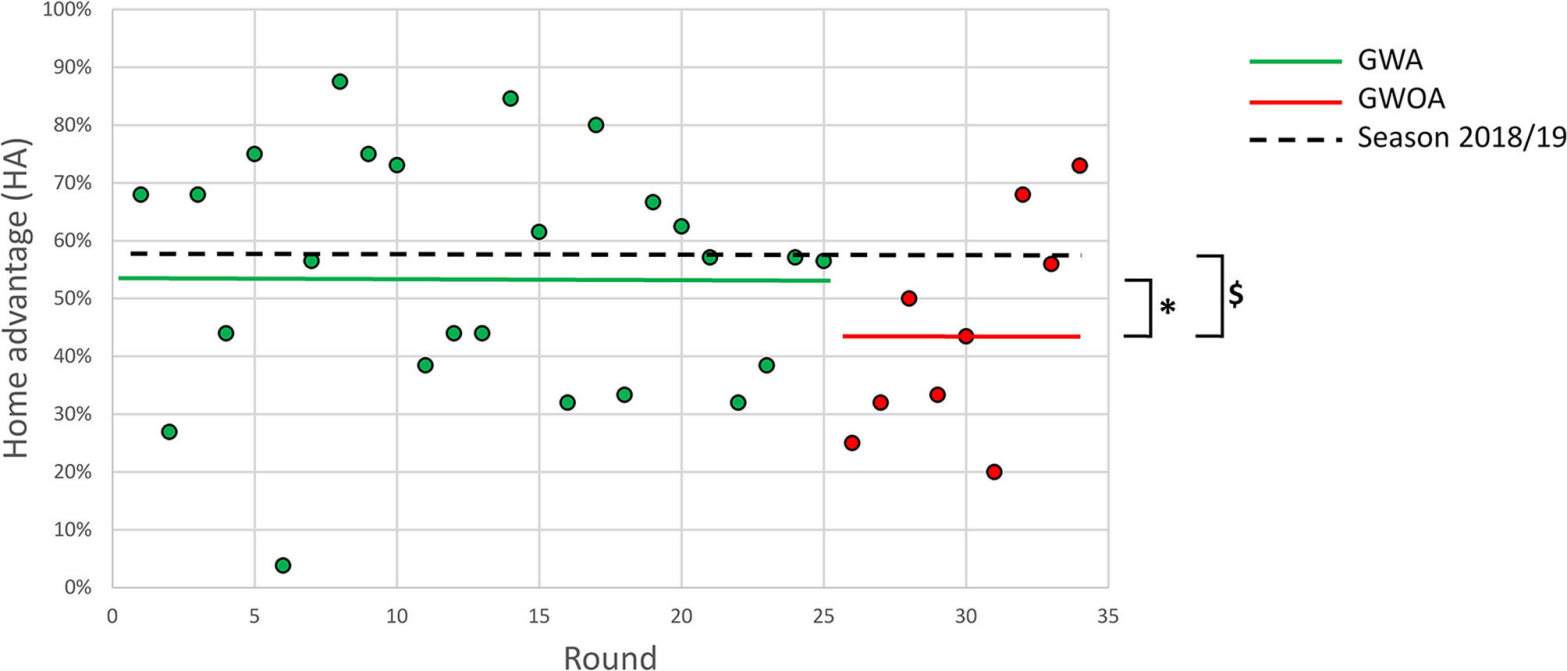
Time course of home advantage (HA) over 34 rounds. Green dots and red dots represent the values for each round in games with (GWA) and without audience (GWOA), respectively. The green and red horizontal lines represent the total HA in GWA and GWOA, respectively. The dashed blacklines represent the total HA over 34 rounds in the season 2018/19. * indicates significant difference to total HA in GWA. $ indicates significant difference to total HA in the season 2018/19.

We found significant differences in HA between GWOA (HA = 44.1%) and GWA (HA = 54.35%) as well as between GWOA and games of the season 2018/19 (HA = 57.63%), *p* = 0.002, and *p* < 0.001, respectively. However, the difference between GWOA and an equal distribution was not significant (*p* = 0.074). Figure 2 shows the time course of the HA for all 34 rounds of the season. No difference in HA was observed between GWA and games from season 2018/19. Table 2 shows a summary of the observed data.

**Table 2:** Summary of the results from the games with (GWA) and without (GWOA) audience of the season 2019/20 and from the full season 2018/19.

	**HW**	**HL**	**HD**	**Home points**	**Away points**	**Total points**	**HA**
GWA (*n* = 223)	96	78	49	337	283	620	54%
GWOA (*n* = 83)	27	36	20	101	128	229	44%
2018/19 (*n* = 306)	138	95	73	487	358	845	58%

*HW, home wins; HL, home losses; HD, draws; HA, home advantage*.

The analysis of the home teams' playing strength of the GWOA revealed that in 53% of the games the home team was better than the guest team according to the final results at the end of the season. The different ranking according to the average number of goals shot by each team during the whole season lead to 48% of better home teams in GWOA. Based on this average number of goals as the rate of occurrences in the Poisson distribution, we computed the predicted numbers of total points for GWA and GWOA as 619.22 and 230.11, respectively. The observed total numbers of points were 620 and 229, respectively (see Table 2). The detailed results of the expected points for each of the 306 games are listed in the Supplementary Material.

From the number of points, we calculated an expected HA of 49.93%. This is not different (*p* = 0.982) to the equal distribution HA = 50% or to the observed HA in GWOA (*p* = 0.078).

A more detailed analysis of the last three rounds of GWOA showed 15 home wins, seven home losses, and five draws, leading to a HA of 65.79%, which differed significantly (*p* = 0.015) from the expected HA = 51.91% calculated via the Poisson distribution. In the last round (34th round of the season, nine games), the observed HA was 73%, again significantly different (*p* = 0.023) from the expected Poisson HA = 51.32%.

In GWA, we counted *n* = 5,344 fouls, 2,595 (48.56%) for the home team and 2,749 (51.45%) for the away team, showing a significant difference to a 50:50 distribution (*p* = 0.035). For the GWOA, we had *n* = 2,111 fouls, 1,067 (50.54%) for the home team and 1,044 (49.46%) for the away team, which was not significantly different (*p* = 0.617) from an equal distribution. The number of average committed fouls per game showed no significant differences between away teams in GWA, home teams in GWOA, and away teams in GWOA (12.327, 12.855, and 12.578, respectively). The average number of committed fouls per game for the home teams in GWA was 11.637, differing significantly (*p* = 0.017) from home teams in GWOA as well as away teams in GWA and GWOA.

The total number of yellow disciplinary cards in the GWA was *n* = 903, 405 (44.85%) for the home and 498 (55.15%) for the away team, respectively. The difference to an expected equal distribution was significant (*p* = 0.002). In the GWOA, we counted *n* = 348, 178 (51.1%) cards for the home and 170 (48.9%) for the away team, respectively, representing no difference to equal distribution (*p* = 0.668), but a significant difference (*p* = 0.018) to the distribution in GWA. The number of red cards differed significantly (*p* = 0.031) from an equal distribution for GWA (14 home and 28 away), but not for GWOA (eight home and seven away). In terms of penalty kicks, we found in GWA as well as in GWOA a home disadvantage (in GWA 22 (43.1%) and in GWOA 8 (38.1 %) penalty kicks for the home team). We did not find any significant differences between GWA and GWOA or to an equal distribution of penalty kicks for home teams and away teams.

## Discussion

The purpose of the study was to analyze the effect of lacking audience on home performance and referee's decisions during the Covid-19 lock-down in the German Bundesliga season 2019/20. In the last nine rounds without audience (GWOA), we did not only observe proportionally fewer home wins than in the previous rounds with audience (GWA), but even more home losses (36) than home wins (27). Hence, the Covid-19 lock-down turned a home advantage of 54.35% into a home disadvantage of 44.10%. Furthermore, the hypothesized omission of a referee's bias during these nine rounds was confirmed by our data with a balanced distribution of penalty cards (home: 51.1%; away: 48.9%), which differed significantly from the previous rounds with audience (home: 55.15%; away: 44.85%). Both during games with and without audience, proportionally fewer penalty kicks were awarded to the home teams (GWA: 43.1%; GWOA: 38.1%).

The lock-down led to an unbalanced schedule for games with and without audience: In the games without audience, each team played either four, five, or six games at home. This had to be considered during the analyses to avoid any false conclusions due to bias. However, the home teams ranked even better in the final season table than the away teams, implying that the chance of winning at home in games without audience was slightly higher just because of the ranking. This makes the finding of a home disadvantage even stronger. However, the final season table results give only an ordinary information about the strength of the teams. To assess the observed HA, we needed another method to calculate an expected HA value for the games after lock-down. Thus, we applied a Poisson distribution for calculating the probability for possible results in each game, taking the average number of goals per game during the whole season as expected rate of occurrences. For a clearly better team, the expected number of points in a game will be close to three, whereas the expected value will be less for an only slightly better team. For equal teams, this value is always larger than one, but depends on the number of average goals/game shot by the two teams. Therefore, this gives more information about the expected points than the ordinary ranking scale obtained by the season's final results. Although the ranking by goals per game showed that in 52% of the games without audience the home team was the better one, the expected HA, calculated via the Poisson distribution, was 49.93% showing no difference to HA = 50% (*p* = 0.983). This implies that the unbalanced partition of all games in games with and without audience did not influence our results significantly. Applying a Poisson distribution is only a very rough estimation. However, the very good prediction of the total points for the games with audience as well as the games without audience (619.22 for 620 Points and 230.11 for 229, resp.) indicates the suitability of using a Poisson distribution. For the advantages and disadvantages of this method, see, e.g., Maher (1982) and Greenhough et al. (2002).

When investigating the influence of the missing audience on the HA, we had the choice between comparing the lock-down phase to the results of the first 25 rounds (GWA) or former seasons. Comparing the HA in games without audience with the data from games with audience had the advantage that we observed the same set of teams. However, the unbalanced schedule in games with audience could also influence the HA. Therefore, we compared the results of the games without audience not only to those with audience in 2019/20 but also to the previous season 2018/19, again finding significant differences. It is notable that the HA before lock-down in 2019/20 was less than in the season before, and also less than reported in literature (e.g., Pollard and Gómez, 2014a with 58.35% for 2006–2012), but not significantly different. This is in accordance with a reported general decline in HA (Pollard, 2008; Peeters and van Ours, 2020).

To the best of our knowledge, so far, a HA below 50% was only reported once based on a meaningful number of games. Pollard and Gómez (2014a) observed a HA of 49.36% in 336 games from the Cayman Islands soccer league (2006–2012). However, even this number is close to an even distribution of points and is difficult to compare with the present results of a high-professional soccer league. Hence, this is the first time a HA below 50% was observed in one of the world's major soccer leagues. The finding of a HA <50% shows that the term “home advantage” is misleading: All values below 50% are a disadvantage. Thus, we suggest the term “home performance” which would fit better to this situation.

Before 2020, only two studies (van de Ven, 2011; Ponzo and Scoppa, 2018) so far could analyze the effect of audience in professional soccer experimentally during same-stadium derbies from the Italian Serie A. In such derbies, familiarity with the stadium and travel fatigue does not play a role, and only crowd support should affect the game outcome. Ponzo and Scoppa (2018) reported a HA of 58.32% in such games. van de Ven (2011) reported a lack of HA in such games and indicated that crowd support is not a necessary factor for home advantage. Calculating HA by the provided data from his Table 2 (van de Ven, 2011) would lead to a HA of 44.53%, i.e., a home disadvantage for the hosting teams in same-stadium derbies. However, this was neither explicitly mentioned nor discussed by the author. Until 2020, van de Ven (2011) was the only author who included games without audience in his home advantage investigations. He reported that home advantage still exists even without audience. However, only 20 games from Italian Serie A and B (season 2007/2008) were included in his study, and a comparison of the data with the present study is difficult because the data analysis is not clearly described.

In 2020, the decrease in HA in soccer games without audience during the Covid-19 pandemic was confirmed by several working papers applying regression models. Home performance, assessed as home wins, point differences between home and away team, or team dominance decreased in the collapsed data from 13 European leagues (McCarrick et al., 2020) and in the first German Bundesliga (Fischer and Haucap, 2020). Interestingly, Fischer and Haucap (2020) did not observe a reduction in home advantage in the second and third German soccer league. They argued that players of the lower leagues might be used to playing in front of smaller crowds. Furthermore, soccer results from individual leagues across the world (Poli et al., 2020) showed a reduction in HA (as % of home wins) in 41 of the 63 leagues studied. The study authors did not observe a decrease of HA in some important leagues like the English Premier League and the Italian Serie A. However, these results must be interpreted with care as team strength was not controlled and the unbalanced playing schedule could have influenced the results. Further analyses of these results are warranted.

Figure 2 shows that the HA increased in the last nine rounds, indicating a recovery of the reduced HA over time. We tested this with a linear regression for the GWOA which showed a significant increase of HA (*R* = 0.7; *p* < 0.05) during this period (see also Figure 2). Fischer and Haucap (2020) confirmed this finding in their analyses of the first three German soccer leagues where they could observe a similar tendency. They hypothesized that players got familiarized to the empty stadiums with time. Since the recovery of the reduced HA is highly influenced by the last three rounds, we made an additional analysis of this period. As they also reflect just a subset of all games, we again checked the possible effects of this unbalanced selection. The expected HA of the last three rounds (27 games), calculated via Poisson distribution, was larger than 50%, but significantly (*p* = 0.015) less than the observed value of HA = 65.79%. This shows that we found a clear home advantage in these three rounds that cannot only be explained by the fact that the home teams were slightly better than the away teams. The last round, consisting of only nine games, featured the largest HA of all nine rounds without audience (HA = 73.08%). The home team won six out of the nine games. The unbalanced sample of games led to a far smaller expected HA of 51.32% which also indicates that the observed significant home advantage is not caused by better home teams. Due to the sudden lock-down, we cannot expect that the psychological effects are constant over the following months. There could rather occur some sort of customization. The HA's rising values in the last rounds could be interpreted as a sign of getting used to the situation. However, if this would already be a steady-state, all other factors besides the crowd would have nearly the same positive effect on the HA. So, we speculate that these peak values would again decrease in time.

The hypothesized omission of a referee's bias was confirmed by our analysis of the fouls and the disciplinary cards. In games with audience, the home teams committed on average fewer fouls than the away teams. This changed in games without audience where we observed an increase in committed fouls for the home team only, which led to a balanced distribution of committed fouls between home and away teams. This was confirmed by Endrich and Gesche (2020), who reported that home teams committed more fouls and were awarded more yellow cards during games without audience in the first two German soccer leagues. These results suggest that the audience makes referees favor the home team rather than discriminate the away team. Contrary, Bryson et al. (2020) reported that during the Covid-19 pandemic fewer cards were given to the away team in their dataset from 23 professional football leagues. Summarized, it appears that the lack of an audience cheering for the home team led to more balanced decisions by the referees. This indicates that the lack of audience reduced the referee's social pressure significantly compared to games with audience (Schwartz and Barsky, 1977; Greer, 1983; Salminen, 1993; Gelade, 2015). These results are somewhat in line with recent reports by Albanese et al. (2020), who found that additional assistance referees helped reduce referee's bias. Interestingly, we could not observe a penalty bias in favor of home teams in games with and without audience in the season 2019/20. This is in contrast to findings by Sutter and Kocher (2004), who reported a clear bias (55 vs. 21 penalties) in favor of home teams in the German Bundesliga in 2000/01. A probable reason for these contrasting results could be the introduction of video assistant systems in 2017/18, which help to re-assess difficult penalty situations with video. The present and previous studies suggest to further support referees on the field by assistant video referees isolated from the home audience. These assistant referees with a wireless connection to the field referees would possibly perceive much less social pressure and therefore make decisions less biased. However, since a more balanced distribution of fouls, disciplinary cards, and penalty kicks can only explain a decrease of HA, but not the observed disadvantage, we can only speculate about the reasons.

Crowd effects are supposed to support the home team, but they also affect the away team (Greer, 1983; Pollard and Gómez, 2014a). On the one hand, the present result with a significant decrease in HA indicates that during the games without audience the lack of cheering and positive gestures, i.e., the social support for the home team (Schwartz and Barsky, 1977), seems to have decreased team performance. On the other hand, also the lack of booing, whistling, and chanting of insults seems to have decreased the distraction for the away team as suggested by Greer (1983). Although these effects for the home and the away team can explain a reduction in HA, these should theoretically not lead to a home disadvantage. Pollard and Gómez (2014a) pointed out that the home advantage is a self-perpetuating phenomenon, meaning that the players' knowledge about the home advantage increases itself. The home team perceives the crowd as “twelfth player” on the field. The sudden absence of the crowd noise and the cheering fans might further demotivate the home team. An additional mental preparation of the home team players regarding the new situation without crowd support might support the players and reduce these negative effects. On the other hand, the away team is also aware of the usual effects of the audience for the home team and thus might be even more motivated in this unusual situation. While the physical familiarity did not change during the lock-down phase, the psychological familiarity did. Furthermore, travel effects should remain the same; however, as travels during the Covid-19 crisis were rare or even impossible, traveling to another city together with team colleagues could have caused positive and motivating effects on the away team.

The results of the present analysis of the first German league cannot simply be transferred to other leagues or types of sport. Analyses of lower German leagues (Fischer and Haucap, 2020) and results of other major leagues (Poli et al., 2020) revealed that the lack of audience did not affect all teams to the same amount. Another limitation of this study is the unbalanced schedule caused by the sudden lock-down and the consequential small number of games without audience. Thus, it was not possible to assess the behavior of single teams playing only 4–5 home games without audience (only one team had 6 home games). The round by round evaluation (Table 3) shows, that the variation of HA is very large such that conclusions on the HA of single teams are statistically not feasible. In addition, the comparison between games with and without audience did not only include a different number of games but also dealt with different constellations of teams, thus the team strengths may have affected the results. Considering the team strength, we applied a model using the Poisson distribution with all its shortcomings. However, as mentioned above, the model gave an excellent prediction of points achieved by the teams.

**Table 3:** Detailed round per round data for the games with (GWA) and without audience (GWOA) in season 2019/20.

	**Round**	**HW**	**HL**	**HD**	**Home points**	**Away points**	**Total points**	**HA (%)**
Games with audience (GWA)	1	5	2	2	17	8	25	68
	2	2	6	1	7	19	26	27
	3	5	2	2	17	8	25	68
	4	3	4	2	11	14	25	44
	5	5	1	3	18	6	24	75
	6	0	8	1	1	25	26	4
	7	3	2	4	13	10	23	57
	8	6	0	3	21	3	24	88
	9	5	1	3	18	6	24	75
	10	6	2	1	9	7	26	73
	11	3	5	1	10	16	26	38
	12	3	4	2	11	14	25	44
	13	3	4	2	11	14	25	44
	14	7	1	1	22	4	26	85
	15	5	3	1	16	10	16	62
	16	2	5	2	8	17	25	32
	17	6	1	2	20	5	25	80
	18	3	6	0	9	18	27	33
	19	6	3	0	18	9	27	67
	20	4	2	3	15	9	24	63
	21*	3	2	3	12	9	21	57
	22	2	5	2	8	17	25	32
	23	3	5	1	10	16	26	38
	24*	3	2	3	12	9	21	57
	25	3	2	4	13	10	23	57
Games without audience (GWOA)	x**	1	1	0	3	3	6	50
	26	1	5	3	6	18	24	25
	27	2	5	2	8	17	25	32
	28	2	2	5	11	11	22	50
	29	3	6	0	9	18	27	33
	30	2	3	4	10	13	23	43
	31	1	6	2	5	20	25	20
	32	5	2	2	17	8	25	68
	33	4	3	2	14	11	25	56
	34	6	2	1	19	7	26	73

*excluding one postponed game that was played without audience. ** two postponed games from round 21 and round 24, played on 11^th^ March and 16^th^ May without audience. HW, home wins; HL, home losses; HD, draws; HA, home advantage.

Limitations in the analyses of the referee bias were the small numbers of penalty kicks and disciplinary cards preventing insight into single games.

A further limitation is the small number of variables: we only looked at the results of the games, the numbers of fouls, disciplinary cards, and penalty kicks. The study would have led to more insight if we had had further information, for example, about the perception of the Covid-19 situation by the players and the trainers.

## Data Availability Statement

The raw data supporting the conclusions of this article will be made available by the authors, without undue reservation.

## Ethics Statement

Ethical review and approval was not required for the study on human participants in accordance with the local legislation and institutional requirements. Written informed consent for participation was not required for this study in accordance with the national legislation and the institutional requirements.

## Author Contributions

MT and ST designed the study, analyzed and interpreted the data, wrote the article, and provided a final approval of the version to be published. All authors contributed to the article and approved the submitted version.

## Conflict of Interest

The authors declare that the research was conducted in the absence of any commercial or financial relationships that could be construed as a potential conflict of interest.
