# Supplementary material for: Covid-19 Has Turned Home Advantage Into Home Disadvantage in the German Soccer Bundesliga
Source: Front Sports Act Living. 2020 Nov 5;2:593499. doi: 10.3389/fspor.2020.593499 (PMC7739793; doi:10.3389/fspor.2020.593499)
Supplement: Supplementary file 2 [file Table_2.DOCX]

*Table B: Expected points due to the calculations using the Poisson distribution for the home teams for each game. The yellow and orange fields mark the GWOA. Green numbers are games won by the home team, red numbers mark the draws and black numbers the games lost by the home team.*

|  | Bayern München | Bayer 04 Leverkusen | VfL Wolfsburg | SC Freiburg | Borussia Mönchen-gladbach | Borussia Dortmund | Eintracht Frankfurt | Werder Bremen | Fortuna Düsseldorf | 1. FC Union Berlin | RB Leipzig | FC Augsburg | TSG Hoffenheim | FC Schalke 04 | Hertha BSC | 1. FC Köln | SC Paderborn 07 | 1. FSV Mainz 05 |
| --- | --- | --- | --- | --- | --- | --- | --- | --- | --- | --- | --- | --- | --- | --- | --- | --- | --- | --- |
| Bayern München |  | 1.99784 | **2.20623** | **2.20623** | **1.91810** | **1.63854** | **2.02986** | **2.30168** | **2.39574** | **2.31748** | **1.68404** | **2.25409** | 2.12612 | **2.36459** | **2.20623** | **2.15820** | **2.38019** | **2.26999** |
| Bayer 04 Leverkusen | 0.82501 |  | 1.62835 | **1.62835** | 1.30472 | **1.02964** | **1.42489** | **1.74650** | **1.86930** | **1.76666** | **1.07211** | **1.68682** | **1.53370** | **1.82788** | 1.62835 | **1.57114** | **1.84853** | **1.70658** |
| VfL Wolfsburg | 0.62842 | 1.14161 |  | **1.41176** | **1.06094** | 0.80821 | 1.17523 | 1.49286 | **1.61883** | **1.51335** | **0.84642** | **1.43260** | **1.28076** | **1.57604** | 1.37415 | **1.31751** | **1.59733** | **1.45249** |
| SC Freiburg | 0.62842 | 1.14161 | **1.37415** |  | **1.06094** | **0.80821** | **1.17523** | 1.49286 | 1.61883 | **1.51335** | **0.84642** | **1.43260** | **1.28076** | 1.57604 | **1.37415** | 1.31751 | 1.59733 | **1.45249** |
| Borussia Mönchen-gladbach | **0.90174** | 1.47999 | **1.71808** | **1.71808** |  | 1.11374 | **1.51523** | **1.83472** | **1.95504** | **1.85453** | 1.15738 | **1.77591** | **1.62402** | **1.91456** | **1.71808** | **1.66129** | **1.93475** | **1.79540** |
| Borussia Dortmund | 1.17550 | **1.77617** | **2.00307** | **2.00307** | **1.69166** |  | **1.81046** | **2.11019** | **2.21795** | **2.12813** | **1.45030** | **2.05651** | 1.91477 | **2.18201** | **2.00307** | **1.94996** | **2.19998** | 2.07438 |
| Eintracht Frankfurt | **0.79441** | **1.35286** | 1.59119 | **1.59119** | 1.26819 | **0.99575** |  | **1.73529** | **1.83338** | 1.73003 | **1.03769** | **1.64983** | **1.49645** | **1.79164** | **1.59119** | 1.53390 | **1.81245** | 1.66966 |
| Werder Bremen | 0.54061 | 1.02147 | 1.24709 | **1.24709** | **0.94427** | 0.70621 | 1.05380 |  | 1.48936 | 1.38432 | 0.74182 | **1.30452** | 1.15593 | 1.44663 | **1.24709** | 1.19172 | 1.46787 | 1.32412 |
| Fortuna Düsseldorf | 0.45568 | 0.89871 | **1.11437** | 1.11437 | 0.82599 | 0.60547 | **0.92933** | 1.22792 |  | **1.24776** | 0.63808 | **1.16999** | **1.02668** | **1.30887** | **1.11437** | **1.06102** | **1.32978** | **1.18904** |
| 1. FC Union Berlin | 0.52623 | 1.00117 | **1.22535** | **1.22535** | **0.92465** | **0.68931** | 1.03325 | 1.34183 | **1.46691** |  | 0.72445 | **1.28253** | 1.13468 | **1.42425** | **1.22535** | **1.17026** | **1.44546** | **1.29412** |
| RB Leipzig | **1.13058** | **1.73010** | **1.95973** | **1.95973** | **1.64503** | 1.35773 | **1.76469** | **2.06878** | **2.17892** | **2.08708** |  | **2.01407** | **1.87015** | 2.14213 | **1.95973** | **1.90581** | **2.16052** | **2.03227** |
| FC Augsburg | **0.58420** | 1.08192 | 1.31136 | **1.31136** | 1.00286 | 0.75710 | **1.11494** | **1.42936** | **1.55520** | **1.44979** | **0.79407** |  | 1.21893 | 1.51237 | **1.31136** | **1.25526** | **1.53368** | **1.38916** |
| TSG Hoffenheim | 0.70325 | **1.23923** | 1.47543 | 1.47543 | 1.15637 | **0.89356** | 1.27361 | **1.59457** | **1.71997** | **1.61504** | 0.93363 | 1.53421 |  | **1.67749** | 1.47543 | **1.41825** | **1.69864** | 1.55416 |
| FC Schalke 04 | 0.48362 | **0.93987** | 1.15921 | **1.15921** | **0.86555** | **0.63885** | **0.97111** | 1.27407 | **1.39809** | **1.29410** | 0.67251 | 1.21552 | **1.07021** |  | **1.15921** | **1.10510** | **1.37677** | **1.23479** |
| Hertha BSC | 0.62842 | **1.14161** | 1.37415 | **1.37415** | **1.06094** | 0.80821 | 1.17523 | **1.49286** | **1.61883** | **1.51335** | 0.84642 | **1.43260** | 1.28076 | **1.57604** |  | 1.31751 | **1.59733** | 1.45249 |
| 1. FC Köln | 0.67317 | **1.20048** | **1.43543** | **1.43543** | 1.11842 | 0.85942 | **1.23459** | **1.55451** | **1.68024** | 1.57501 | 0.89877 | **1.49413** | 1.34134 | **1.63760** | 1.43543 |  | **1.65883** | **1.51407** |
| SC Paderborn  07 | 0.46960 | 0.91932 | 1.13687 | 1.13687 | 0.84578 | 0.62213 | **0.95025** | 1.25109 | **1.37465** | **1.27103** | 0.65527 | 1.19284 | **1.04850** | 1.33240 | 1.13687 | 1.08312 |  | 1.20802 |
| 1. FSV Mainz 05 | 0.56960 | 1.06185 | 1.29010 | 1.29010 | 0.98338 | 0.74011 | **1.09465** | **1.40778** | **1.53350** | 1.42817 | 0.77663 | 1.34794 | 1.19805 | **1.49070** | **1.29010** | **1.23422** | **1.51571** |  |
