# Supplementary material for: Covid-19 Has Turned Home Advantage Into Home Disadvantage in the German Soccer Bundesliga
Source: Front Sports Act Living. 2020 Nov 5;2:593499. doi: 10.3389/fspor.2020.593499 (PMC7739793; doi:10.3389/fspor.2020.593499)
Supplement: Supplementary file 3 [file Table_3.DOCX]

*Table A: Results of all games in the season 2019/20. The yellow and orange fields mark the GWOA. Green numbers indicate home wins, red numbers indicate draws, and black numbers indicate home losses.*

|  | Bayern München | Bayer 04 Leverkusen | VfL Wolfsburg | SC Freiburg | Borussia Mönchengladbach | Borussia Dortmund | Eintracht Frankfurt | Werder Bremen | Fortuna Düsseldorf | 1. FC Union Berlin | RB Leipzig | FC Augsburg | TSG Hoffenheim | FC Schalke 04 | Hertha BSC | 1. FC Köln | SC Paderborn 07 | 1. FSV Mainz 05 |
| --- | --- | --- | --- | --- | --- | --- | --- | --- | --- | --- | --- | --- | --- | --- | --- | --- | --- | --- |
| Bayern München |  | 1:2 | **2:0** | **3:1** | **2:1** | **4:0** | **5:2** | **6:1** | **5:0** | **2:1** | **0:0** | **2:0** | 1:2 | **5:0** | **2:2** | **4:0** | **3:2** | **6:1** |
| Bayer 04 Leverkusen | 2:4 |  | 1:4 | **1:1** | 1:2 | **4:3** | **4:0** | **2:2** | **3:0** | **2:0** | **1:1** | **2:0** | **0:0** | **2:1** | 0:1 | **3:1** | **3:2** | **1:0** |
| VfL Wolfsburg | 0:3 | 0:2 |  | **2:2** | **2:1** | 0:2 | 1:2 | 2:3 | **1:1** | **1:0** | **0:0** | **0:0** | **1:1** | **1:1** | 1:2 | **2:1** | **1:1** | **4:0** |
| SC Freiburg | 1:3 | 0:1 | **1:0** |  | **1:0** | **2:2** | **1:0** | 0:1 | 0:2 | **3:1** | **2:1** | **1:1** | **1:0** | 4:0 | **2:1** | 1:2 | 0:2 | **3:0** |
| Borussia Mönchengladbach | **2:1** | 1:3 | **3:0** | **4:2** |  | 1:2 | **4:2** | **3:1** | **2:1** | **4:1** | 1:3 | **5:1** | **1:1** | **0:0** | **2:0** | **2:1** | **2:0** | **3:1** |
| Borussia Dortmund | 0:1 | **4:0** | **3:0** | **1:0** | **1:0** |  | **4:0** | **2:2** | **5:0** | **5:0** | **3:3** | **5:1** | 0:4 | **4:0** | **1:0** | **5:1** | **3:3** | 0:2 |
| Eintracht Frankfurt | **5:1** | **3:0** | 0:2 | **3:3** | 1:3 | **2:2** |  | **2:2** | **2:1** | 1:2 | **2:0** | **5:0** | **1:0** | **2:1** | **2:2** | 2:4 | **3:2** | 0:2 |
| Werder Bremen | 0:1 | 1:4 | 0:1 | **2:2** | **0:0** | 0:2 | 0:3 |  | 1:3 | 0:2 | 0:3 | **3:2** | 0:3 | 1:2 | **1:1** | 6:1 | 0:1 | 0:5 |
| Fortuna Düsseldorf | 0:4 | 1:3 | **1:1** | 1:2 | 1:4 | 0:1 | **1:1** | 0:1 |  | **2:1** | 0:3 | **1:1** | **2:2** | **2:1** | **3:3** | **2:0** | **0:0** | **1:0** |
| 1. FC Union Berlin | 0:2 | 2:3 | **2:2** | **2:0** | **2:0** | **3:1** | 1:2 | 1:2 | **2:0** |  | 0:4 | **2:0** | 0:2 | **1:1** | **1:0** | **2:0** | **1:0** | **1:1** |
| RB Leipzig | **1:1** | **1:1** | **1:1** | **1:1** | **2:2** | 0:2 | **2:1** | **3:0** | **2:2** | **3:1** |  | **3:1** | **3:1** | 1:3 | **2:2** | **4:1** | **1:1** | **8:0** |
| FC Augsburg | **2:2** | 0:3 | 1:2 | **1:1** | 2:3 | 3:5 | **2:1** | **2:1** | **3:0** | **1:1** | **1:1** |  | 1:3 | 2:3 | **4:0** | **1:1** | **0:0** | **2:1** |
| TSG Hoffenheim | 0:6 | **2:1** | 2:3 | 0:3 | 0:3 | **2:1** | 1:2 | **3:2** | **1:1** | **4:0** | 0:2 | 2:4 |  | **2:0** | 0:3 | **3:1** | **3:0** | 1:5 |
| FC Schalke 04 | 0:3 | **1:1** | 1:4 | **2:2** | **2:0** | **0:0** | **1:0** | 0:1 | **3:3** | **2:1** | 0:5 | 0:3 | **1:1** |  | **3:0** | **1:1** | **1:1** | **2:1** |
| Hertha BSC | 0:4 | **2:0** | 0:3 | **1:0** | **0:0** | 1:2 | 1:4 | **2:2** | **3:1** | **4:0** | 2:4 | **2:0** | 2:3 | **0:0** |  | 0:5 | **2:1** | 1:3 |
| 1. FC Köln | 1:4 | **2:0** | **3:1** | **4:0** | 0:1 | 1:3 | **1:1** | **1:0** | **2:2** | 1:2 | 2:4 | **1:1** | 1:2 | **3:0** | 0:4 |  | **3:0** | **2:2** |
| SC Paderborn 07 | 2:3 | 1:4 | 2:4 | 1:3 | 1:3 | 1:6 | **2:1** | 1:5 | **2:0** | **1:1** | 2:3 | 0:1 | **1:1** | 1:5 | 1:2 | 1:2 |  | 1:2 |
| 1. FSV Mainz 05 | 1:3 | 0:1 | 0:1 | 1:2 | 1:3 | 0:4 | **2:1** | **3:1** | **1:1** | 2:3 | 0:5 | 0:1 | 0:1 | **0:0** | **2:1** | **3:1** | **2:0** |  |
